# Huangqi Gegen decoction ameliorates alcohol-induced cognitive dysfunction via attenuating oxidative stress and enhancing blood-brain barrier integrity in rats through the Keap1-Nrf2/HO-1 signaling pathway

**DOI:** 10.22038/ijbms.2024.77415.16737

**Published:** 2024

**Authors:** Yang Qiao, Qing Yuan, Zhen Liu

**Affiliations:** 1 Department of Traditional Chinese Medicine, Baotou Central Hospital, Baotou, Inner Mongolia, China; 2 State Key Laboratory of Component-based Chinese Medicine, Tianjin Key Laboratory of Traditional Chinese Medicine Pharmacology, Tianjin University of Traditional Chinese Medicine, Tianjin, China

**Keywords:** Alcohols, Blood-Brain Barrier Cognitive, dysfunction HuangQi, Oxidative stress, Pueraria

## Abstract

**Objective(s):**

Chronic alcohol abuse causes cognitive deficits. Huangqi Gegen Decoction (HGD), a traditional Chinese herbal formula comprising Huangqi and Gegen, has been documented for its therapeutic efficacy in the treatment of alcoholic liver injury. However, its potential neuroprotective effects against alcohol-induced brain injury remain unexplored. This study aims to evaluate the neuroprotection of HGD on alcohol-induced cognitive dysfunction and the associated mechanism.

**Materials and Methods:**

Wistar rats were orally administered 50% ethanol for 10 weeks, followed by treatment with HGD at doses of 16, 32, or 64 mg/kg/day for an additional 6 weeks. The spatial learning and memory abilities of rats were assessed through the Morris Water Maze experiment. The pathological condition in the hippocampus was assessed using H&E and Nissl staining. Tight junction proteins, oxidative stress, and inflammation cytokines were measured by IF, ELISA, PCR, and western blot. The mRNA and protein expression of Keap1, Nrf-2, HO-1, and NQO-1 were tested by PCR and western blot.

**Results:**

Results showed that HGD effectively mitigated cognitive dysfunction and pathological changes in alcohol-induced rats while enhancing the expression of ZO-1, Occludin, and Claudin-5. Furthermore, HGD effectively mitigated oxidative stress by reducing levels of ROS and MDA, while elevating levels of SOD, CAT, and GSH-PX in brain tissue. Moreover, HGD significantly suppressed microglial activation and down-regulated expressions of IL-1β, IL-6, and TNF-α. Mechanistically, HGD remarkably up-regulated the expression of Nrf-2, HO-1, and NQO-1 while down-regulating Keap1 expression.

**Conclusion:**

These findings suggest that HGD may be a promising therapeutic agent for alleviating alcohol-induced cognitive dysfunction.

## Introduction

Alcohol (ethanol) is the most commonly abused substance worldwide and has posed significant public health challenges in recent years (1). Prolonged excessive consumption of alcohol represents a crucial risk factor for cerebrovascular disease and brain damage. The lipophilic nature of alcohol facilitates its passage across the blood-brain barrier (BBB), thereby augmenting BBB permeability and triggering oxidative stress and inflammatory responses, ultimately resulting in significant impairments to learning and memory (2, 3). However, due to insufficient understanding of the underlying mechanisms and a lack of corresponding drug options, there is still a lack of good interventions for the treatment of chronic alcoholic cognitive dysfunction.

Long-term alcohol consumption can induce a significant accumulation of reactive oxygen species (ROS) in the brain, leading to an elevation in oxidative stress markers and inflammatory factors (4, 5). This process triggers the activation of both oxidative stress and neuroinflammatory responses within the brain, ultimately resulting in cognitive decline (6). Numerous studies have demonstrated that chronic alcohol consumption reduces the activities of antioxidant enzymes such as superoxide dismutase (SOD) and glutathione peroxidase (GSH-PX), while concurrently increasing Malondialdehyde (MDA) activity. Consequently, this impairs cellular capacity to eliminate free radicals, thereby causing damage to brain nerve cells (7, 8). In addition to inducing oxidative stress, ROS can also impede cognitive functions associated with learning, memory, neuronal survival, and synaptic plasticity by promoting the release of inflammatory cytokines such as interleukin (IL)-6, IL-1β, and tumor necrosis factor (TNF)-α (9). Therefore, inhibition of oxidative stress and neuroinflammation in the brain is a promising measure to improve alcoholic cognitive dysfunction (10).

BBB is a crucial structure for maintaining the homeostasis and functionality of the central nervous system, effectively preventing the infiltration of toxins and pathogens into the brain through stringent regulation of internal environmental components (11). Studies have demonstrated that chronic alcohol consumption induces oxidative stress and neuroinflammation, leading to impairment in BBB integrity in mice by down-regulating the expression of tight junction proteins (Claudin-5, Occludin, and ZO-1) between endothelial cells (12-14). A recent study has reported a class of drugs that exhibit potential for ameliorating alcohol-induced cognitive dysfunction (15-16). The investigation revealed that this improvement may be closely associated with anti-inflammatory and antioxidant properties, preservation of BBB integrity, as well as regulation of synaptic plasticity.

Huangqi Gegen Decoction (HGD) is a traditional Chinese medicine prescription listed in the third volume of “Zhengzhihuibo”, which has been widely used in the treatment of diabetes mellitus and stroke sequelae (17). This prescription mainly takes Huangqi and Gegen as a drug pair, and the effective components are total flavonoids and total saponins (18). Huangqi, the dried root of leguminous plants *Astragalus mongolica *or* Astragalus membranaceus*, has anti-inflammatory, antioxidant, inhibition of apoptosis, maintaining the integrity of the BBB, and neuroprotective effects (19-20). Gegen, derived from the legume kudzu, is the dried root of this plant. Pharmacological studies have shown that Gegen contains isoflavones, puerarin, triterpene saponins, and other chemical components, which have antioxidant effects and can alleviate hangover symptoms and other physiological effects (21-22). These studies suggest that both Huangqi and Gegen exhibit significant antioxidant effects and can reduce BBB permeability, indicating their potential efficacy in ameliorating alcohol-induced cognitive dysfunction. However, the impact and mechanism of HGD on alcohol-induced brain injury have not been previously reported. Therefore, this study aims to establish a rat model of chronic alcohol injury and investigate the potential cognitive improvement effects of HGD in these rats while also elucidating its underlying mechanisms. Ultimately, our findings may provide novel intervention strategies for preventing and treating alcoholic cognitive dysfunction.

## Materials and Methods

***Reagents and drugs***

Huangqi (*A. membranaceus*) and Gegen (*Pueraria lobata*) were purchased from Guangdong Yifang Pharmaceutical Co. LTD (GuangDong, China). Nissl stain kit (#G1430) and H&E kit (#C0105) were purchased from Boster (Wuhan, China). Antibodies for IBA-1(ab178680), Claudin-5 (ab15106), Occludin (ab167161), ZO-1 (ab59720), and β-actin (ab8227) were purchased from Abcam (Massachusetts, USA). Nrf-2 (A21176), NQO-1 (A23486), HO-1 (A19062), and Keap1 (A25175) were purchased from ABclonal Biotech Co., Ltd (Wuhan, China).

***Animals and treatments***

**Figure 1:**
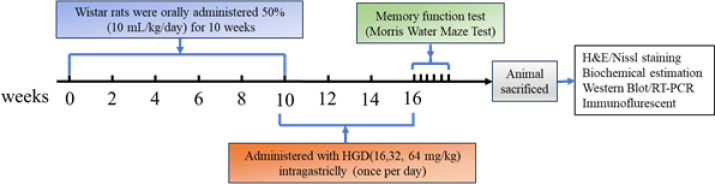
Schematic representation of experimental procedure

Adult male Wistar rats weighing 250-300 g were procured from Sibeifu (Beijing) Biotechnology Co., Ltd and housed in a temperature-controlled room with a relative humidity of 55%±5% and a light/dark cycle of 12/12 hr. This experiment was approved by the Animal Ethics Committee of Baotou Medical College, Inner Mongolia. All animal manipulations were conducted in accordance with the Arrive guidelines for reporting *in vivo* experiments involving animals. The rats were randomly divided into five groups: (1) normal control group (Control), in which the rats had free access to tap water; (2) model group treated with ethanol (Alcohol), in which the rats received 50% ethanol (10 ml/kg/day, intragastrically) for 10 weeks (23); (3-5) three groups treated with different doses of HGD combined with ethanol: HGD group at high dosage (64 g/kg/day, HGD-H), HGD group at medium dosage (32 g/kg/day, HGD-M), or HGD group at low dosage (16 g/kg/day, HGD-L). All experiments were conducted using a blinded, randomized, and controlled design to collect data. The schematic diagram illustrating the experimental design of alcohol-induced rats and HGD treatment is presented in Figure 1.

***Morris water maze test***

The Morris Water Maze (MWM) experiment is a behavioral assessment aimed at evaluating the spatial learning and memory capabilities of animals. The swimming trajectory of rats is automatically tracked using an image monitoring system, while the cognitive abilities of rats are assessed by recording parameters such as escape latency period, number of target quadrant crossings, and time spent in each experimental trial. Initially, the rats underwent training to locate submerged platforms in an opaque circular pool filled with water. The starting positions were randomly assigned from four directions: east, west, south, and north. The time taken by each rat to discover the underwater platform was recorded in seconds. If a rat took more than 90 sec during the initial training session to locate the platform, assistance was provided to guide it toward the target location. Subsequently, the rat rested on the platform for 10 sec before being removed and dried. Each subject underwent four daily training sessions for 4 consecutive days with a 30-minute interval between sessions. On day 5, the platform is removed and the animals are subsequently immersed in the water from a position opposite to their initial platform quadrant. The image monitoring system automatically tracks and records the swimming trajectory, speed, and time taken by the rats to reach the platform. Specialized image acquisition and analysis software is employed for data analysis (24). Additionally, weekly weighing of rats in each group during a 6-week administration period was conducted to assess the impact of HGD on body weight.

***Nissl and H*** ***&*** ***E staining***

Paraffin-embedded brain sections (3 μm) were subjected to hematoxylin-eosin (H&E) and Nissl staining using a modified protocol provided by the manufacturer. Pathological changes were observed and documented under a Leica microscope, while Image J software was utilized for quantification of intact neurons (25).

***Detection of ROS generation***

ROS testing was performed as previously described (26). Briefly, DCFH-DA (10 μmol/l) was added to the diluted brain tissue homogenate and incubated at 37 ^°^C for 45 min. Subsequently, cellular esterase deacetylated DCFH-DA into a non-fluorescent compound which was further oxidized by ROS to generate 2’-7’ dichlorofluorescein (DCF). The fluorescence of DCF, a fluorescent compound, was measured using a fluorescent enzyme spectrometer with an excitation wavelength set at 485 nm and an emission wavelength at 525 nm. Quantification of reactive oxygen species (ROS) levels in brain tissue was achieved through the use of a DCF standard curve.

***Measurement of oxidative stress and inflammation factors***

The levels of MDA, SOD, CAT, and GSH-PX in brain tissue were quantified using a commercially available kit (Nanjing Jiancheng Bioengineering Institute, China) following the manufacturer’s instructions (27). The levels of inflammatory cytokines IL-1β (CSB-E08055r), IL-6 (CSB-E04640r), and TNF-α (CSB-E11987r) were detected using ELISA assay kits from Wuhan Huamei Biotechnology Co., Ltd (China)(28).

***Immunofluorescence (IF)***

The paraffin-embedded brain sections (3 μm) were deparaffinized using xylene and subsequently rehydrated through a graded series of ethanol. Following antigen retrieval, the slices were permeabilized with 0.25% Triton-X 100 and blocked to prevent non-specific binding. Subsequently, the tissue slices were incubated overnight at 4 ^°^C with primary antibodies targeting IBA-1, and Claudin-5 (dilution 1:200). On the following day, after triple washing in PBS to remove any unbound primary antibodies, a fluorescent secondary antibody (dilution 1:1000) was applied for 1 hr. Subsequently, the nuclei were stained with DAPI for 5 min and visualized using a fluorescence microscope (Zeiss, Germany)(29).

***RNA isolation and quantitative real-time PCR (RT-PCR)***

Total RNA in brain tissue was extracted using TRIzol® reagent (Invitrogen/Life Technologies, Carlsbad, CA, USA). The complementary DNA (cDNA) was synthesized using cDNA reverse transcription kits (Applied Biosystems, Foster City, USA). RT-PCR was performed to verify the differential expression of selected genes by using a CFX Connect Real-Time System (BIO-RAD, USA) with SYBR® Green PCR Master Mix reagent kits (Applied Biosystems, Foster City, USA). The specific primer pairs (Shanghai Sangon Biotech Co., Ltd., Shanghai, China) are listed in Table 1. The mRNA levels were normalized to the level of β-actin, and the fold change of the threshold cycle (Ct) value of the treated group relative to the control sample was calculated in accordance with the following equation: fold change=2 ^(−ΔΔCt)^. All samples were analyzed in triplicate (30).

***Western blot***

The brain tissue was homogenized in lysis solution, followed by separation of resulting protein lysates using SDS/PAGE gels. Subsequently, the proteins were transferred onto PVDF membranes and incubated overnight at 4 ^°^C with appropriate primary antibodies against IBA-1, ZO-1, Occludin, Claudin-5, Nrf2, Keap-1, NQO-1, HO-1, and β -actin at a dilution of 1:1000. Following this step, the PVDF membranes were incubated with a secondary antibody (diluted to 1:10000) for 1 hr prior to visualization using the Super ECL Detection Reagent kit (Yeasen, China)(31).

***Statistical analysis***

The results were presented as mean±SD and statistical analysis was conducted using IBM SPSS Statistics 22.0 or Prism 9.0 software packages. Differences between groups were evaluated through one-way analysis of variance (ANOVA) with Tukey’s multiple comparison test. For the MWM data, repeated measures (RM)-ANOVA analysis was conducted over the preceding four days. The level of significance was set at *P*<0.05.

## Results

***HGD improves the ability of learning and memory in ethanol-induced rats***

**Figure 2:**
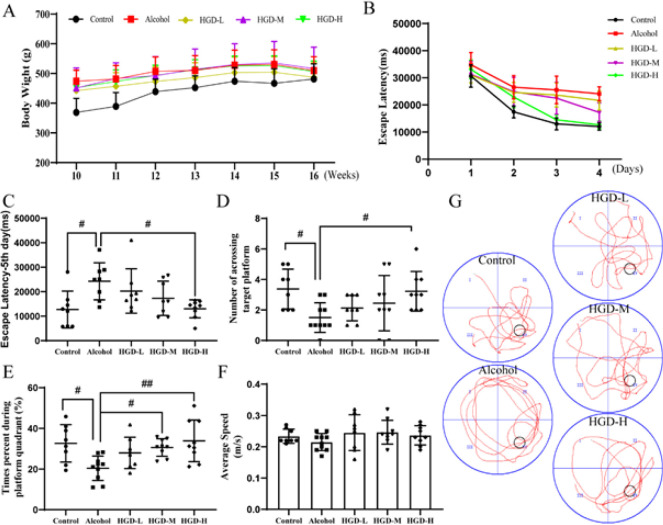
Effects of Huangqi Gegen Decoction (HGD) on body weight and spatial learning and memory in rats with ethanol injury

Throughout the experimental period, there were no significant differences observed in body weight changes among the groups (Figure 2A). We employed the Morris water maze test (MWM) to evaluate the effect of HGD on cognitive function in alcohol-induced spatial cognitive deficit rats (Figure 2B-G). Following a consecutive 4-day MWM study, the escape latency on day 5 was significantly prolonged in the alcohol group compared to the normal control group (*P*=0.0203). Concurrently, administration of HGD resulted in a dose-dependent reduction in escape latency on day 5, with significant shortening observed in the HGD-H group compared to the alcohol group (*P*=0.0198) (Figure 2B-C). In the spatial search experiment on day 5, the high-dose HGD group exhibited a significant increase in the number of platform crossings compared to the alcohol group (*P*=0.0451)(Figure 2D). Additionally, rats in the HGD-M and HGD-L groups spent significantly more time in the goal platform quadrant than those in the alcohol group (*P*=0.0474 and *P*=0.0043)(Figure 2E). No significant differences in swimming speed were observed between the groups, indicating that the drug itself, rather than swimming speed, was responsible for the variations in cognitive dysfunction-related measures (Figure 2F). Representative plots of spatial exploration trajectories of rats in each group are depicted in Figure 2G.

**Table 1:** Primer sequences used for RT-PCR quantification of IL-6, IL-1β, TNF-α and Keap1, Nrf2, HO-1, NQO-1

Gene	Primer Pair (5’-3’) F，forward; R，reverse
Rat -β-Actin	F 5’-GTAAAGACCTCTATGCCAACA-3’ R 5’-GGACTCATCGTACTCCTGCT-3’
Rat -IL-6	F 5’- CCAGAGATACAAAGAAATGATGG 3’ R 5’- ACTCCAGAAGACCAGAGGAAA - 3’
Rat -IL-1β	F 5’- CTCCATGAGCTTTGTACAAGG -3’ R 5’- TGCTGAT GTACCAGTTGGGG -3’
Rat -TNF-α	F 5’- CGGGGTGATCGGTCCCCAAAG -3’ R 5’- GGAGGGCGTTGGCGCGCTGG -3’
Rat -Keap1	F 5’-CACCAGGGCAGGATCTAC-3’ R 5’-TTGCTTCCGACAGGGTTC-3’
Rat -Nrf2	F 5’-CTGCTGCCATTAGTCAGTCG-3’ R 5’-GCCTTCAGTGTGCTTCTGGT-3’
Rat -HO-1	F 5’-CAGAGTTTCTTCGCCAGAGG-3’ R 5’-TGAGTGTGAGGACCCATCG-3’
Rat -NQO-1	F 5’-TCCAGAAACGACATCACAGG-3’ R 5’-AGCTACAATATCCGGGCTCA-3’

Primers used are all synthesized by Shanghai Bioengineering Co., Ltd

(A) Body weight changes. (B) Escape latency in the spatial learning phase. (C) Escape latency on the 5th day. (D) Times of crossing the platform. (E) Proportion of time spent in the goal plateau quadrant. (F) Swimming speed. (G) Representative graph of the spatial exploration trajectories of rats in each group. Data are expressed as mean±SD (n=8 per group). ^#^*P<*0.05, ^##^*P<*0.01

**Figure 3:**
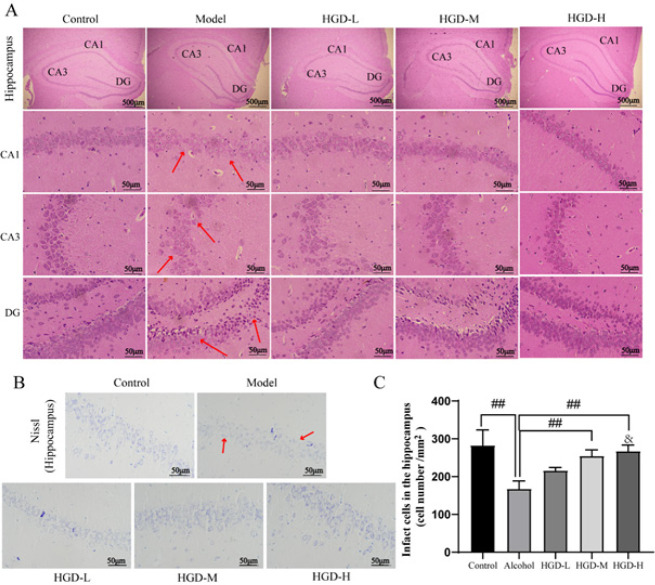
Huangqi Gegen Decoction (HGD) promotes characteristic pathology in ethanol-induced rats

(A) Representative observation of hippocampus sections with H&E staining, Scale bar, 200 μm. (B) Nissl staining in the hippocampus (Bar=100 μm). (C) Statistics of the number of Nissl bodies. (n=4 per group). ^#^*P<*0.05, ^##^*P<*0.01; ^&^ *P<*0.05 vs HGD-L group

**Figure 4:**
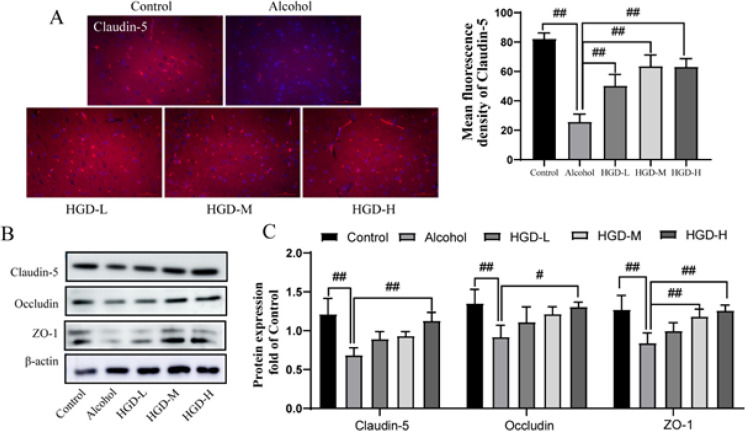
Huangqi Gegen Decoction (HGD) significantly increased the expression of ZO-1, Claudin-5, and Occludin in ethanol-induced brain injury rats

(A) Immunofluorescence staining of Claudin-5 from sections in each group (n=4, the scale bars are 100 μm). (B-C) Western blot bands and quantification of Claudin-5, Occludin, and ZO-1 in each group (n=4 per group). ^#^*P<*0.05, ^##^*P<*0.01

**Figure 5:**
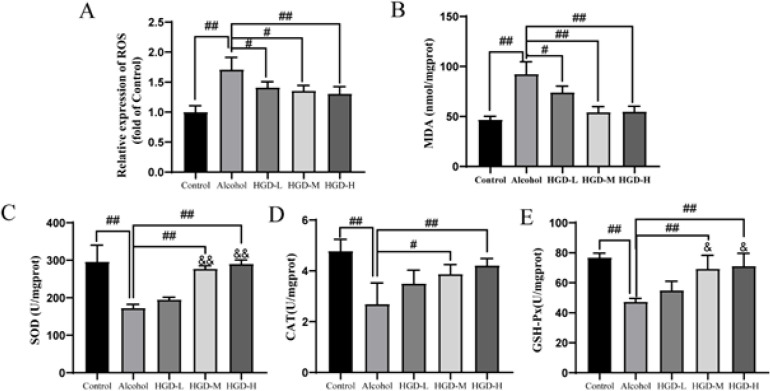
Effects of huangqi Gegen Decoction (HGD) on oxidative stress in the brain of alcohol-injured rats

(A) ROS contents; (B) MDA contents; (C) SOD activity; (D) CAT activity; and (E) GSH-PX activity in rat’s brain. Data are represented as mean±SD (n=4 per group). ^#^*P<*0.05, ^##^*P<*0.01, ^&^ *P<*0.05, and ^&&^ *P<*0.01 vs HGD-L group

ROS: Reactive oxygen species; MDA: Malondialdehyde; SOD: Superoxide dismutase; GSH-PX: Glutathione peroxidase

**Figure 6:**
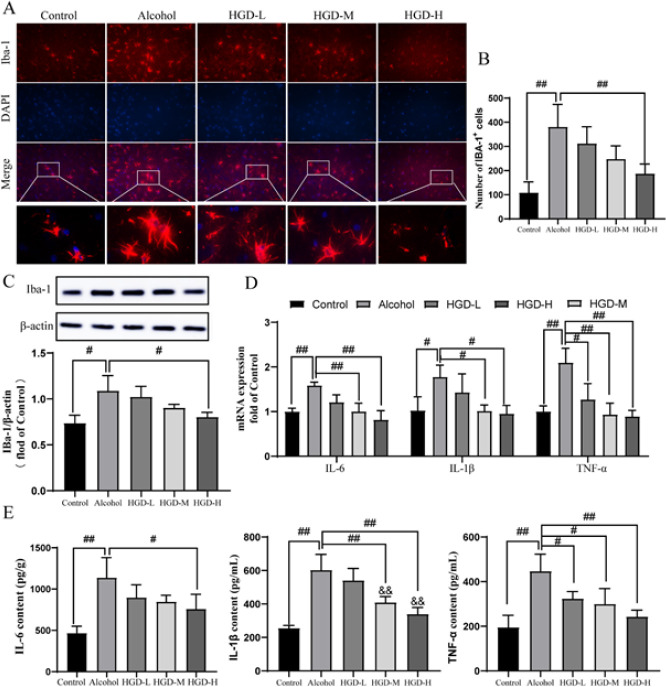
Effects of Huangqi Gegen Decoction (HGD) on inflammation factors in the brain of alcohol-injured rats

(A-B) IBA-1 -positive microglia from sections in each group (the scale bars are 50 μm). (C) Western blot bands and quantification of GFAP and IBA-1 in each group. (D) mRNA expression of IL-6, IL-1β, TNF-α in each group; (E) IL-6, IL-1β, and TNF-α contents. Data are represented as mean±SD (n=4 per group). ^#^*P<*0.05, ^##^*P<*0.01. ^&&^*P<*0.01 vs HGD-L group

**Figure 7:**
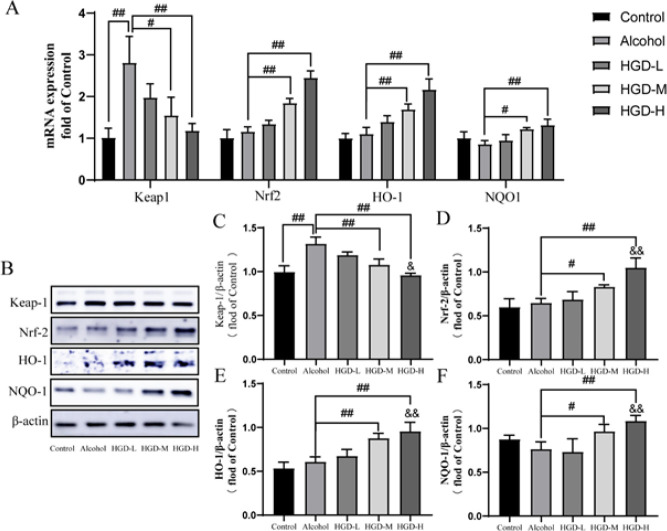
Huangqi Gegen Decoction (HGD) increases in the mRNA and protein expressions of Nrf-2, HO-1, and NQO-1 and decreases in the level of Keap-1 in ethanol-induced rats

(A) Expressions of Keap1, Nrf-2, HO-1, and NQO-1 mRNA were measured with RT-PCR. (B) Expressions of Keap1, Nrf-2, HO-1, and NQO-1 proteins were measured with western blot. (C-F) Densitometric analysis was performed using the Quantity One software. Data are presented as mean±SD from 4 experiments. ^#^*P<*0.05, ^##^*P<*0.01. ^&^*P<*0.05, ^&&^*P<*0.01 vs HGD-L group

**Figure 8:**
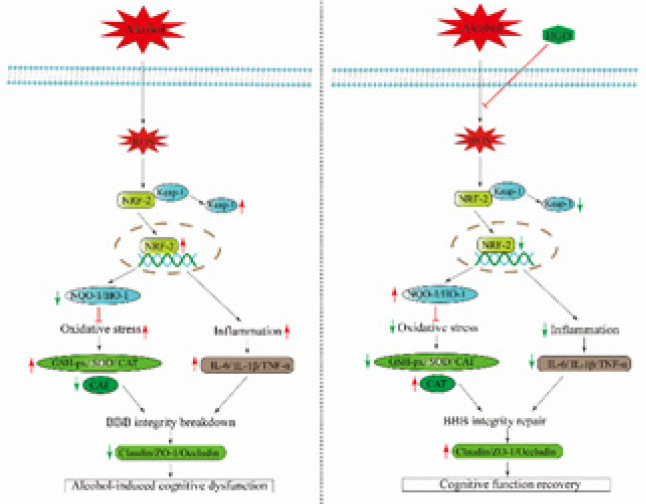
Schematic diagram summarizes the possible mechanism of Huangqi Gegen Decoction (HGD) in ameliorating alcohol-induced cognitive dysfunction

***HGD promotes characteristic pathology in ethanol-induced rats***

The well-organized arrangement of neurons in the hippocampus serves as the structural foundation for learning and memory. Pathological alterations in hippocampal neurons following treatment were assessed using HE staining. In alcohol-injured rats, a significant reduction in neuronal density was observed in the CA1, CA3, and DG regions of the hippocampus, accompanied by a more dispersed neuronal arrangement compared to the normal control group. However, treatment with HGD resulted in remarkable improvements in pathological lesions within the CA1, CA3, and DG areas, characterized by increased nerve density and a more compact organization (Figure 3A). The number of Nissl bodies in hippocampal neurons of each treatment group was determined using Nissl staining. The results revealed a significant increase in the number of neurons in the hippocampus of rats treated with HGD-M and HGD-H compared to the alcohol group (*P*=0.0007 and *P*=0.0002). In addition, the HGD-H group significantly increased the number of neurons compared with the HGD-L group (*P*=0.0439)(Figure 3B, 3C).

***HGD decreased tight junction protein expression in ethanol-induced brain injury rats***

The integrity of the BBB structure ensures the performance of the brain’s internal environment and cognitive functions, and TJ proteins Claudin-5, Occludin, and ZO1 are functional proteins of the BBB structure. As shown in Figure 4A, compared with the control group, the fluorescence expression of Claudin-5 in the brain tissue of the alcohol group was significantly reduced (*P*<0.0001), indicating that the TJ structure was damaged, while the expression level of Claudin-5 was significantly increased after the administration of HGD-L, HGD-M, and HGD-H (*P*=0.0004,* P*<0.0001, and* P*<0.0001). Western blot results further showed that the protein expression of Claudin-5, Occludin, and ZO-1 in the alcohol-injured group decreased significantly compared with the control group (*P*=0.0002, *P*=0.0058, and *P*=0.0013). However, the ZO-1 protein expression was significantly increased in the HGD-M group (*P*=0.0094), and the protein expression levels of Claudin-5, Occludin, and ZO-1 were significantly increased in the HGD-H group (*P*=0.001, *P*=0.0131, and *P*=0.0017)(Figure 4C-D).

***HGD ameliorated oxidative stress in rats with ethanol-induced brain injury***

Alcohol-induced overproduction of ROS leads to the induction of oxidative stress and inflammatory responses, thereby mediating damage to brain cells. MDA, a direct byproduct of ROS, can serve as a reliable biomarker for assessing levels of oxidative stress. As depicted in Figure 5A-B, the expression levels of both ROS and MDA were significantly elevated in the alcohol group compared to those in the normal control group (*P*<0.0001 and *P*<0.0001), indicating severe oxidative stress within the rat brain. However, there were notable reductions observed in the expression levels of both ROS and MDA following intervention with HGD-L (*P*=0.0318 and *P*=0.0187), HGD-M (*P*=0.0101 and *P*<0.0001), and HGD-H groups (*P*=0.0034 and *P*<0.0001). Compared with the HGD-L group, MDA expression showed significant decrease in both HGD-M (*P*=0.0103) and HGD-H groups (*P*=0.0132). Moreover, compared to the normal control group, alcohol-induced brain injury rats exhibited significantly reduced activities of SOD, CAT, and GSH-PX in brain hippocampal tissue (*P*<0.0001, *P*<0.0001, and* P*<0.0001). However, these antioxidant enzymes displayed significantly enhanced activities following HGD-M (*P*<0.0001, *P*=0.0389, *and* *P*=0.0015) and HGD-H intervention (*P*<0.0001, *P*=0.0069, *and* *P*=0.0007). In addition, compared with the HGD-L group, the activities of SOD and GSH-PX were significantly enhanced in HGD-M (*P*<0.0001 and *P*=0.0383) and HGD-H groups (*P*<0.0001 and* P*=0.0174) (Figure 5C-E). These findings suggest that HGD effectively enhances the antioxidant capacity of alcohol-damaged rats.

***HGD ameliorated the inflammation response in rats with ethanol-induced brain injury***

The excessive generation of ROS induced by alcohol consumption further elicits the onset of an inflammatory response. The activation and proliferation of microglia are intimately associated with inflammation. Immunofluorescence results revealed a significant increase in the number of IBA-1-positive microglia in the alcohol-injured group (*P*=0.0002). Compared to the alcohol-injured group, HGD treatment significantly reduced the number of IBA-1-positive cells in rat brains (*P*=0.0044) (Figure 6A-B). Consistent with these findings, western blot analysis revealed that protein expression levels of IBA-1 were significantly increased in brain tissue from alcohol rats (*P*=0.0108) but were markedly decreased following HGD treatment (*P*=0.0317)(Figure 6C). Moreover, the mRNA and protein expression levels of pro-inflammatory cytokines IL-6 (*P*=0.0055 and *P*<0.0001), IL-1β (*P*=0.0472 and *P*<0.0001) and TNF-α (*P*=0.0026 and *P*<0.0001) were significantly up-regulated in the brains of rats with alcohol-induced injury, while the mRNA and protein expressions of TNF-α were significantly decreased in HGD-L group (*P*=0.0173, *P*=0.0443). The mRNA expressions of IL-6 (*P*=0.0056, *P*=0.0007), IL-1β (*P*=0.0437, *P*=0.0286), TNF-α (*P*=0.0017, *P*=0.0136) and protein expressions of IL-6 (*P*=0.0437, *P*=0.009), IL-1β (*P*=0.0021, *P*<0.0001), TNF-α (*P*=0.0136, *P*=0.0008) were significantly decreased in HGD-M and HGD-H groups. In addition, compared with the HGD-L group, the protein expression of IL-1β was significantly decreased in the HGD-M (*P*=0.04) and HGD-H (*P*=0.0015) groups (Figure 6D-E). These findings suggest that HGD exerts a mitigating effect on chronic alcohol-induced inflammatory response.

***Effects of HGD on Keap1-Nrf2/HO-1 pathway-related proteins in rats with ethanol-induced brain injury***

To further elucidate the mechanism underlying the improvement of cognitive function in alcohol-impaired rats by HGD, we employed RT-PCR and western blot techniques to assess the expression levels of proteins associated with the Keap1-Nrf2/HO-1 pathway in rat brain tissues. The results demonstrated a significant increase in the mRNA and protein expressions of Keap1 in the alcohol group compared to the control group (*P*=0.0136, *P*=0.0008). Additionally, while there was an increase in Nrf-2, NQO-1, and HO-1 expressions in the brain tissue of the alcohol group, this difference did not reach statistical significance (*P*>0.05). The mRNA expressions of Nrf-2 (*P*=0.0009, *P*<0.0001), HO-1 (*P*=0.035, *P*=0.0068), NQO-1 (*P*=0.0095, *P*<0.0001) and protein expressions of Nrf-2 (*P*=0.0402, *P*<0.0001), HO-1 (*P*<0.0001, *P*<0.0001), NQO-1 (*P*=0.0466, *P*=0.0014) were significantly up-regulated in HGD-M and HGD-H groups. While mRNA and protein expression of Keap1 were down-regulated in HGD-M (*P*=0.0186, *P*=0.0036) and HGD-H (*P*=0.0034, *P*=0.0001) groups. In addition, compared with the HGD-L group, the protein expression of Nrf-2 (*P*=0.0001), HO-1 (*P*=0.0007), and NQO-1 (*P*=0.0005) was significantly increased, while the protein expression of Keap1 (*P*=0.0047) was significantly decreased in HGD-H group (Figure 7A-F).

## Discussion

Epidemiological studies have demonstrated a positive correlation between chronic alcohol consumption and an elevated risk of cerebral damage (32). The neurotoxic effects of alcohol and its metabolites on the brain can result in cerebral ischemia and hypoxia, induce oxidative stress, compromise the integrity of the blood-brain barrier, and contribute to cognitive dysfunction (33). Reducing alcohol consumption has been demonstrated as an effective strategy for mitigating alcohol-induced brain injury. However, achieving abstinence remains a formidable challenge due to the addictive nature of alcohol. Therefore, we conducted a study investigating the ameliorative effects of HGD on alcohol-induced brain injury in rats.

Chronic alcohol consumption has been associated with acceleration of cognitive decline, as evidenced by studies reporting significant memory impairment and other cognitive deficits in mice following continuous alcohol treatment (34). The MWM experiment is a behavioral assessment designed to evaluate animals’ spatial learning and memory capabilities (35). In this study, we initially employed MWM to evaluate cognitive function in rats and observed that HGD significantly mitigated memory impairment induced by alcohol exposure. Compared to the control group, the alcohol-induced group exhibited an increase in escape latency and a decreased number and longer time of platform crossings, indicating impaired learning and memory function in rodents due to chronic alcohol consumption. Additionally, the successful establishment of an alcohol-induced cognitive impairment model in rats was also demonstrated. The HGD treatment effectively ameliorates these behavioral changes, suggesting that ethanol exposure impairs the spatial memory and spatial search abilities of rats, while HGD can ameliorate spatial learning and memory deficits induced by chronic alcohol consumption in rats.

The hippocampus, a pivotal brain region, plays a crucial role in cognition. The optimal arrangement and abundance of neurons within the hippocampus serve as the structural foundation for facilitating learning and memory. Chronic alcohol consumption induces characteristic pathological alterations in the hippocampus of rats, including a reduction in cell number and volume, cellular deformation, and mild nuclear pyknosis, ultimately leading to cognitive dysfunction (36). This finding is consistent with the observed decline in learning, memory, and cognitive function among patients suffering from chronic alcoholism (37). The H&E staining results revealed a significant reduction in the local neuronal density within the CA1, CA3, and DG regions of the hippocampus in the alcohol-injured rats compared to the normal control group. Additionally, there was an observed dispersion in the arrangement of neurons. However, upon administration of HGD, gradual recovery of pathological damage was observed throughout various areas of the hippocampus in alcohol-injured rats. This recovery was characterized by increased nerve density and a more compact arrangement structure. Additionally, Nissl staining revealed a significant increase in the number of neuronal Nissl bodies in the hippocampus following the administration of HGD.

Alcohol-induced elevation of ROS and oxidative stress can harm the endothelial barrier function, thereby compromising the BBB integrity through modulation of intercellular tight junction protein expression and increased permeability, consequently exacerbating cognitive deficits in the brain (14, 38). In the BBB structure, TJ proteins including Claudin-5, Occludin, and ZO-1 play a crucial role in maintaining TJ integrity of brain endothelial cells. These proteins effectively seal the intercellular space between adjacent endothelial cells, forming a highly selective permeable barrier for circulating molecules (39). Ethanol has been demonstrated to dose-dependently decrease the expression of TJs (claudin-5, occludin, and ZO-1) in HCMEC/D3 cells, resulting in disruption of the BBB structure and impairment of its function (40). In order to further investigate the impact of HGD on BBB injury in alcohol-induced brain injury rats, we assessed the expression levels of Claudin-5, Occludin, and ZO-1, which are closely associated with BBB tight junction proteins. Our findings demonstrate that HGD intervention effectively restored the reduced expression of Claudin-5, Occludin, and ZO-1 induced by alcohol exposure, suggesting that HGD may enhance the structural integrity of the BBB in rats with alcohol-induced brain injury.

Long-term chronic alcoholism can lead to the accumulation of ROS in the brain, resulting in elevated levels of peroxides within the body. This process disrupts the balance between cellular antioxidants and oxidative stress by altering the activity of intracellular antioxidant enzymes (41). Research has demonstrated that prolonged alcohol consumption can decrease antioxidant enzyme activity (SOD, CAT, and GSH-PX) while increasing MDA within the brain (42). The results of the experiment demonstrated that, compared to the model group, the HGD treatment group exhibited increased levels of antioxidant enzymes SOD, CAT, and GSH-PX in brain tissue. Additionally, ROS and MDA content decreased. Moreover, the excessive generation of ROS further stimulates microglia, thereby eliciting an inflammatory response that exacerbates neuronal damage in alcohol-induced brain injury (43). Our study demonstrated a significant increase in the number of IBA-1-positive microglia in the brain tissue of rats with alcohol-induced injury. However, treatment with HGD resulted in a reduction in the number of positive cells, as confirmed by protein expression results from IBA-1 blot experiments. The results demonstrate that following HGD intervention, there was a significant decrease in mRNA and protein expression of IL-1β, IL-6, and TNF-α. These findings suggest that HGD treatment exerts potent antioxidant and anti-inflammatory effects while conferring neuroprotection against ethanol-induced cognitive dysfunction.

The Keap1-Nrf2/HO-1 signaling pathway is widely recognized as a pivotal endogenous antioxidant stress pathway, playing a crucial role in the regulation of antioxidant defense and inflammation suppression (44). Kelch-like epichlorohydrin-associated protein 1 (Keap1)/nuclear factor E2 associated factor 2 (Nrf2) constitutes an intrinsic redox-responsive module for cellular protection gene expression, ensuring the maintenance of homeostasis (45). Under the influence of oxidative stress caused by ROS, Nrf2 dissociates from Keap1 and translocates into the nucleus, where it activates endogenous antioxidants such as heme oxygenase 1 (HO-1) and quinone oxidoreductase 1 (NQO-1). This process effectively mitigates cellular damage induced by oxidative stress (46). Enhanced expression of the Keap1-Nrf2/HO-1 pathway through pharmacological or gene therapeutic approaches has been demonstrated as a promising strategy for intervening in the pathophysiology of oxidative stress (47). Therefore, activation of the Keap1-Nrf2/HO-1 signaling pathway via antioxidant therapy is likely to represent a safe and effective treatment modality for preventing, delaying, or treating alcohol-induced cognitive dysfunction. The results of this study demonstrate a significant increase in mRNA and protein levels of Nrf2, HO-1, and NQO-1 in the HGD group, accompanied by a reduction in Keap-1 mRNA and protein levels. These findings suggest that HGD may confer neuroprotection against ethanol-induced brain injury through activation of the Keap1-Nrf2/HO-1 signaling pathway.

## Conclusion

In summary, HGD effectively mitigates chronic alcohol-induced brain injury through inhibition of oxidative stress, suppression of glial cell activation and proliferation, and enhancement of BBB integrity. The underlying mechanism can be attributed to its activation of the Keap1-Nrf2/HO-1 pathways (the proposed mechanism of HGD is shown in Figure 8). These findings not only demonstrate the role and potential mechanism of HGD in treating alcohol-induced brain injury but also expand the clinical application of HGD, providing a novel strategy for managing this condition.
